# Merkel Cell Carcinoma of the Hand

**DOI:** 10.1016/j.jhsg.2026.101081

**Published:** 2026-06-19

**Authors:** Brooklyn VanDerWolde, Victoria Durkin, Kelly Jerstad, James Breit, Robert E. Van Demark

**Affiliations:** ∗ University of South Dakota Sanford School of Medicine, Sioux Falls, SD; † Department of Graduate Medical Education, University of South Dakota Sanford School of Medicine, Sioux Falls, SD; ‡ Internal Medicine Department, University of South Dakota Sanford School of Medicine, Sioux Falls, SD; § Surgery Department, University of South Dakota Sanford School of Medicine, Sioux Falls, SD; ‖ Department of Orthopedics, University of South Dakota Sanford School of Medicine, Sioux Falls, SD

**Keywords:** Malignant skin cancer, Merkel cell polyomavirus, Merkel cell tumor, Neuroendocrine tumor, Single-fraction radiation therapy

## Abstract

Merkel cell carcinoma (MCC) is a rare and aggressive neuroendocrine skin cancer, typically involving the head, neck, and rarely extremities. MCC of the hand is rare, and early diagnosis is critical to optimizing patient outcomes. We report a case of MCC of the hand in a 79-year-old woman with a 1-month history of a left-hand mass. A punch biopsy of the lesion confirmed the diagnosis of MCC. Treatment consisted of wide local excision with 1.5 cm margins, split-thickness skin grafting, a negative sentinel node biopsy, followed by a single treatment of adjuvant radiation therapy. This case emphasizes the importance of early detection of MCC in atypical locations and highlights the challenges in diagnosis and management. Early recognition and aggressive treatment of MCC is critical to improving patient outcomes. This case also discusses a change in adjuvant radiation therapy protocols for MCC.

Merkel cell carcinoma (MCC) is a rare and aggressive neuroendocrine skin cancer, typically involving the head, neck and rarely extremities. MCC of the hand is rare, and early diagnosis is critical to optimize patient outcomes. There is an estimated incidence of approximately 3,000 new cases annually in the United States, with a recurrence rate of 55% to 79%.^1^, ^2^, ^3^, ^4^ Merkel cell polyomavirus (MCPyV) causes 80% of MCC tumors in the United States, and chronic ultraviolet exposure is responsible for 20% of tumors.^1^ Other risk factors for MCC are age >50 years, male sex, fair skin, history of multiple skin cancers, and chronic immunosuppression.^1^^,^^3^ The average age at diagnosis is 69 years. MCC is typically found in sun-exposed areas, especially the head and neck, and rarely the hands and wrist, which can make the diagnosis challenging.^1^^,^^3^ Given its rapid growth, high recurrence rates, and potential for metastasis, early diagnosis and treatment are critical.^1^^,^^3^ Approximately one-third of cases present with metastatic disease to the lymph nodes. MCC has a mortality rate of 25% to 36%, which is higher than malignant melanoma.^1^, ^2^, ^3^^,^^5^

We report a case of MCC of the hand in a 79-year-old woman with a 1-month history of a left-hand mass. A punch biopsy of the lesion confirmed the diagnosis of MCC. Treatment consisted of wide local excision with 1.5 cm margins, split-thickness skin grafting, a negative sentinel node biopsy (SLNB), followed by a single treatment of adjuvant radiation therapy. This case emphasizes the importance of early detection of MCC in atypical locations and highlights the challenges in diagnosis and management. Early recognition and aggressive treatment of MCC is critical to improving patient outcomes. This case also discusses a change in adjuvant radiation therapy protocols for MCC.

### Informed consent

Informed written consent was obtained from the patient and she agreed to submission of this project. She agreed that her participation was completely voluntary, and she had the right to withdraw her permission at any time.

## Case Report

**Figure 1:**
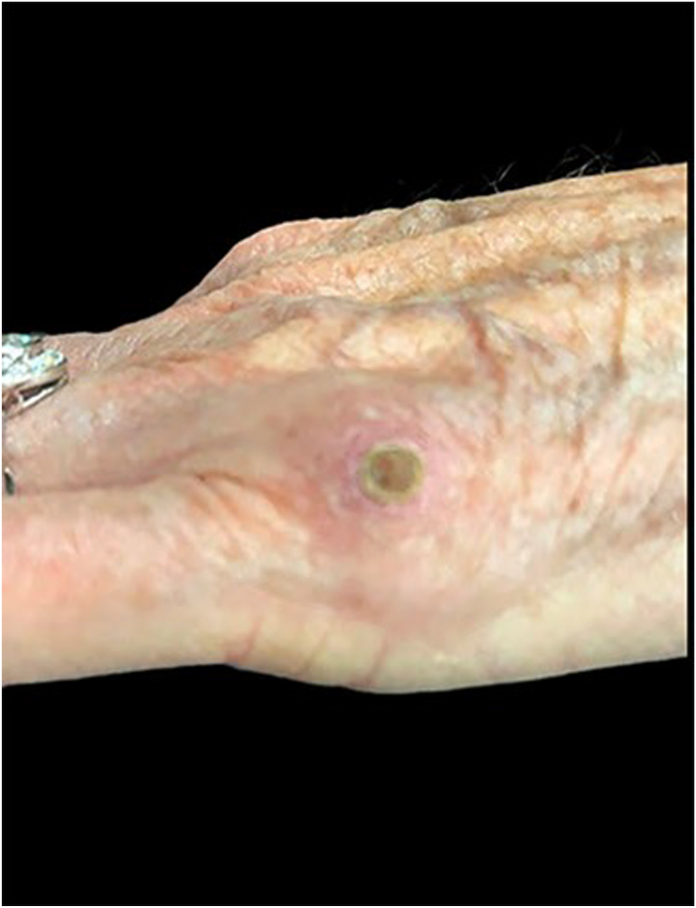
Clinical photograph of Merkel cell lesion following punch biopsy.

**Figure 2:**
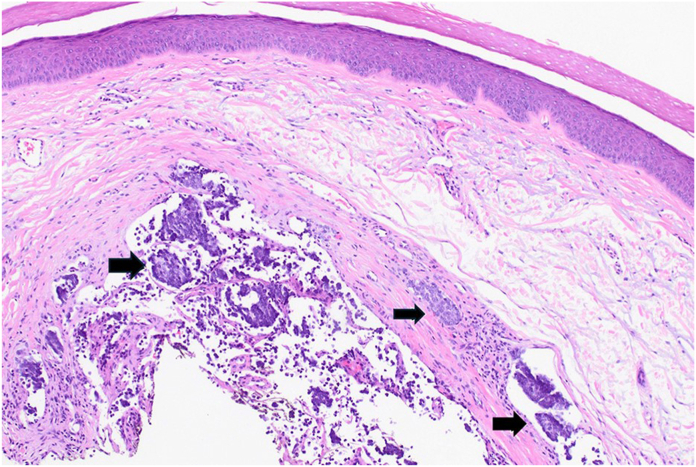
Photomicrograph showing nests of basaloid cells within superficial dermis (black arrows) (Hematoxylin-eosin stain; magnification x 10).

**Figure 3:**
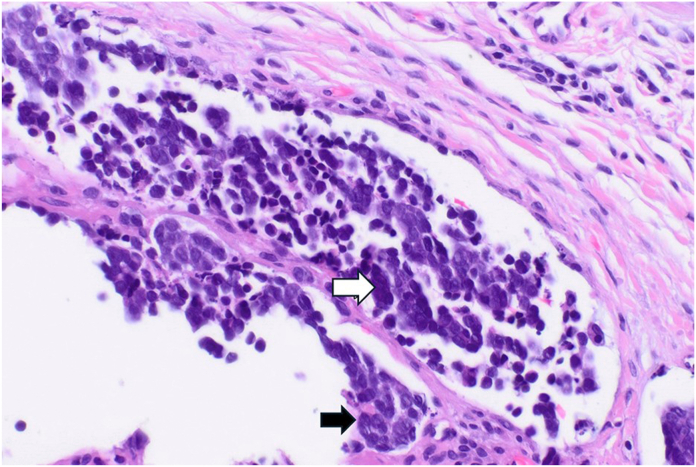
Photomicrograph showing nests of basaloid cells with high nuclear-to-cytoplasm ratio, apoptotic cells (black arrow) and nuclear molding (white arrow) (Hematoxylin-eosin stain; magnification x 40).

**Figure 4:**
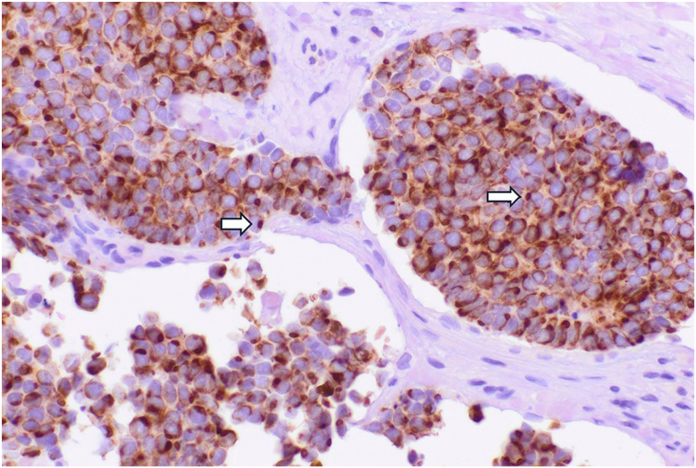
Photomicrograph showing positive immunohistochemical staining for CK20 in a paranuclear dot-like pattern (white arrows) (magnification x 40).

**Figure 5:**
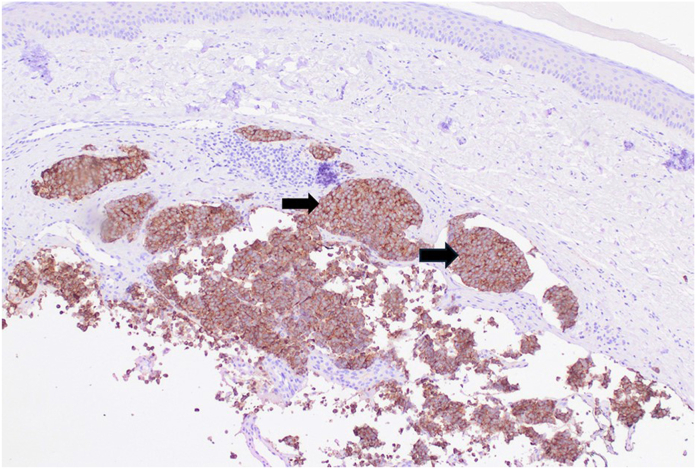
Photomicrograph showing positive immunohistochemical staining for synaptophysin (black arrows) (magnification x 10).

A 79-year-old woman presented with a 1-month history of a painless, rapidly enlarging mass on the dorsum of her left hand. There was no history of trauma or prior skin lesions. The hand lesion measured approximately 1.5 × 1.5 cm at presentation (Fig. 1). Radiographs of the hand revealed a previous wrist fusion. A shave biopsy was performed by her dermatologist and showed dermal infiltration of atypical small, blue cells with scant cytoplasm, irregular nuclei, and frequent mitotic figures (Figs. 2 and 3). Immunohistochemistry demonstrated strong positivity for CK20 along with synaptophysin and chromogranin (Figs. 4 and 5). Staining was negative for TTF-1, ruling out metastatic small cell lung carcinoma. These microscopic findings were consistent with MCC.

**Figure 6:**
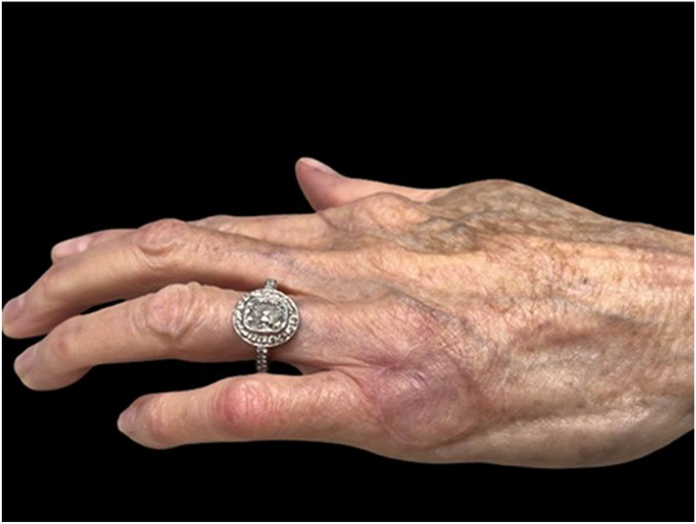
One-year postoperative clinical photograph.

One month following the biopsy, she underwent a wide local excision of the hand lesion with 1.5 cm margins. Two hours before the procedure, the nuclear medicine team injected the patient with Lymphoseek radioactive dye (1.08 mCi of technetium-99m tilmanocept). Prior to the mass excision under general anesthesia, the dermis surrounding the mass was injected with methylene blue. The lesion was encapsulated and did not involve the underlying extensor tendon paratenon. Using the thigh as a donor site, the operative site was covered with a split-thickness skin grafting. Surgical pathology margins were clear with 8 mm margins. Using a hand-held gamma detection probe, SLNB was performed. Three sentinel nodes were identified. The three nodes were negative. The first two lymph nodes were superficial (level I) basin, with radiotracer counts of 5,400 and 14,393, respectively. The deeper node in the level II basin demonstrated the highest tracer uptake at 29,837 counts and was consistent with the dominant sentinel node. Following node removal, residual background counts measure 368, representing a greater than tenfold reduction and confirming adequate sentinel node harvest. No methylene blue uptake was seen in the excised nodes. Final pathology demonstrated clear margins with a minimum margin of 8 mm, and all sentinel nodes were negative for metastatic disease. Her postoperative course was uneventful with complete healing of the skin graft. She was evaluated by the oncology tumor board. Based on her negative SLNB and clear surgical margins, the oncology team believed that a positron emission tomography (PET) scan was not needed. Merkel cell virus (MCPyV) testing was performed. The serum Merkel oncoprotein antibody titer level was < 74 STU, which is considered as a negative titer. Based on current guidelines, radiation therapy to the operative site was recommended. Radiation therapy was delayed for skin graft healing. Three months following surgery, her wound was well-healed, and she had no pain. Following consultation with the radiation oncology team, she received a single fraction of orthovoltage (8 Gy) to the operative site. Serum Merkel cell antibody testing was negative. At her 1-year follow-up examination, she was doing well with no sign of tumor recurrence (Fig. 6).

## Discussion

Merkel cell carcinoma of the hand is rare, with rare cases reported in literature. Its unusual location can lead to misdiagnosis as a benign lesion such as a cyst, lipoma, or more common skin cancers, delaying appropriate management.^3^^,^^6^^,^^7^ The clinical presentation of MCC is nicely summarized by the term AEIOU: Asymptomatic/no tenderness, Expanding rapidly (3 months), Immunosuppression, Older that 50, and Ultraviolet skin exposure.^7^

MCPyV is a member of the *Polyomaviridae* family of DNA viruses. The natural hosts of these viruses are mammals and birds. The virus is acquired in childhood and is commonly present in the skin of healthy individuals. The MCPyV virus is part of normal skin flora and is a well-known risk factor for MCC.^2^^,^^3^

**Figure 7:**
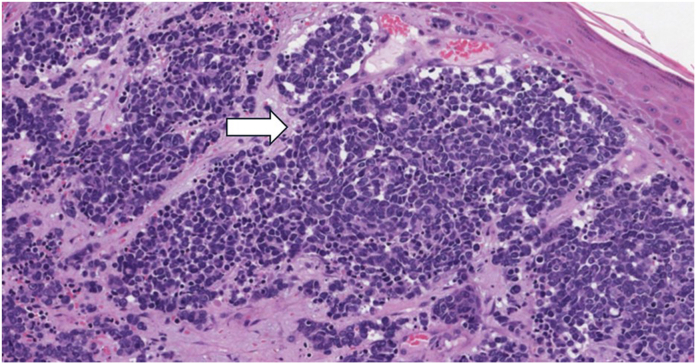
Sheets and nests of basaloid cells with nuclear molding (white arrow).

**Figure 8:**
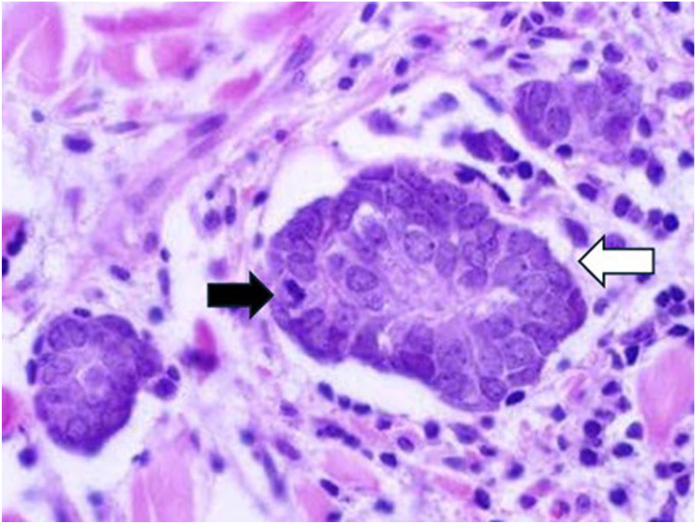
Nests of basaloid cells (white arrow) with nuclear molding, stippled chromatin, and frequent mitotic figures (black arrow).

MCC is a member of the small blue round cell tumors group. The hallmark microscopic findings include sheets and nests of basaloid cells with nuclear molding, stippled chromatin and frequent mitotic figures (Figs. 7 and 8).^1^^,^^2^^,^^6^ Three types of MCC have been described. These include small cell, trabecular, and intermediate. MCC tumors release cytoskeletal keratins (type I or type II). The presence of CK20 keratin positivity in a dot-like pattern is the hallmark finding in diagnosing MCC.^2^^,^^8^

Serum MCPyV antibody testing has been used as a method of following disease recurrence. A considerable rise in the antibody titer may be seen with persistent or recurrent MCC.^3^

Treatment of MCC typically involves a multimodal approach, including surgical excision of the tumor, SLNB, adjuvant radiation therapy, and systemic therapy (including immunotherapy with checkpoint inhibitors) for metastatic disease.^1^, ^2^, ^3^^,^^6^ Surgery is the recommended treatment for both primary site and metastatic lymph node MCC involvement.^1^^,^^4^^,^^6^

Current National Comprehensive Care Network (NCCN) guidelines recommend surgery for localized disease and radiotherapy for both local and regional spread.^6^^,^^7^ Surgery includes wide local excision (WLE) with 1–2 cm margins and sentinel node lymph biopsy (SLNB).^1^, ^2^, ^3^^,^^6^^,^^7^

Mohs surgery has also been used for treating MCC. It is useful in clinical situations where traditional WLE guidelines would endanger underlying soft tissue structures, such as the head and neck and dorsum of the hand.^1^^,^^3^ Several authors have reported using extensor retinaculum subcutaneous fat, paratenon, and fascia as the deep margin for tumor resection.^3^^,^^7^ Based on these case reports, nonamputation resection appears to provide adequate control of the disease.^3^^,^^6^

In a case series from the Mayo Clinic, the relapse rate following Mohs surgery was comparable to WLE.^3^

As part of the surgical treatment, sentinel lymph node biopsy is a critical component of staging, as MCC has a high propensity for early lymphatic spread. SLNB is useful in determining the extent of the disease and is used to guide treatment options.^3^^,^^4^^,^^6^^,^^7^

Because MCC is a radiosensitive tumor, the use of adjuvant radiotherapy, regardless of tumor size, location or nodal involvement, improves survival rates.^6^^,^^9^^,^^10^ Adjuvant radiation therapy is considered for cases with both negative and positive margins or nodal involvement. The literature recommends starting radiotherapy no later than 8 weeks postoperative to minimize the risk of recurrence.^9^

Conventional fractionated postoperative radiation therapy has been traditionally considered for MCC patients but is associated with acute toxicity and requires daily treatments for several weeks.^10^ In the past, traditional radiotherapy consisted of 50 Gy in 25 fractional treatments.^1^^,^^6^^,^^10^ The current NCCN guidelines for adjuvant radiotherapy include the following: 50–55 Gy with clinically negative margins, 56–60 Gy for microscopically positive margins, and 60–66 Gy for grossly positive margins or unresectable lesions.^6^

Recent studies support the use of single-fraction radiation therapy as a viable alternative to conventional fractionated postoperative radiation therapy for localized MCC to maintain local control while minimizing toxicity.^10^ This treatment option features higher doses in fewer treatment fractions (eg, 8 Gy in 1–3 fractions).^1^^,^^3^^,^^7^^,^^10^

Systemic chemotherapy has been used for metastatic MCC or lesions that cannot be treated with surgery or radiation.^3^ The treatment protocols for small cell lung cancer have been adapted for treating MCC. There are no studies to evaluate the use of systemic chemotherapy in MCC patients.^3^^,^^6^

Because of the high recurrence rate, MCC patients need to be followed closely. The NCCN guidelines recommend a complete physical examination, including lymph node evaluation, every 3–6 months for 2 years. Following that, patients should be seen every 6–12 months.^7^

This case highlights MCC of the hand as a rare but aggressive malignancy that requires early recognition and prompt treatment. Increased awareness of MCC in atypical locations is crucial to reducing delays in diagnosis and improving prognosis. Given its potential for rapid progression and metastasis, clinicians should maintain a high index of suspicion for MCC when evaluating rapidly growing nodules on the hand. Early biopsy, accurate staging with SLNB, and appropriate treatment can significantly improve patient outcomes. Recent studies have shown the effectiveness of radiation therapy in treating MCC. This case highlights the option of using single-fraction radiation therapy, which simplifies the postoperative radiation treatment protocol.

## Conflicts of Interest

No benefits in any form have been received or will be received related directly to this article.
